# Association of trimethylamine oxide and its precursors with cognitive impairment: a systematic review and meta-analysis

**DOI:** 10.3389/fnagi.2024.1465457

**Published:** 2024-10-04

**Authors:** Caiyi Long, Zihan Li, Haoyue Feng, Yayi Jiang, Yueheng Pu, Jiajing Tao, Rensong Yue

**Affiliations:** ^1^Hospital of Chengdu University of Traditional Chinese Medicine, Chengdu, China; ^2^Chengdu University of Traditional Chinese Medicine, Chengdu, China

**Keywords:** trimethylamine oxide, TMAO, circulating concentration, cognitive impairment, meta-analysis

## Abstract

**Objectives:**

The role of trimethylamine oxide (TMAO) in patients with cognitive impairment remains controversial. This study aimed to assess the association between TMAO and its precursors and the prevalence of cognitive impairment.

**Methods:**

PubMed, Embase, and Web of Science databases were searched for studies that met the inclusion criteria from their inception to 14 September 2024, and references were manually searched to identify any additions. Odds ratio (OR) was assessed by random-effects modeling, subgroup analyses to identify potential sources of heterogeneity, and the Newcastle-Ottawa Scale (NOS) and the Agency for Healthcare Research and Quality (AHRQ) Inventory for qualitative evaluation.

**Results:**

Nine studies involving 82,246 participants were included in the analysis. Meta-analyses suggested that elevated TMAO levels were strongly associated with an increased risk of cognitive impairment (OR: 1.39, 95% confidence interval [95%CI]: 1.09–1.77, *p* < 0.05, I^2^:60%), and consistent results were obtained across all subgroups examined and sensitivity analyses. However, in the TMAO dose–response meta-analysis and TMAO precursor meta-analyses, the results were not significantly different (dietary choline: OR: 0.93, 95%CI: 0.78–1.10, *p* = 0.385, I^2^:68%, plasma choline: OR: 0.65, 95%CI: 0.41–1.02, *p* = 0.063, I^2^:76%, plasma betaine: OR: 0.74, 95%CI: 0.52–1.05, *p* = 0.094, I^2^:61%).

**Conclusion:**

We found that high TMAO concentrations were positively associated with the risk of cognitive impairment. TMAO is expected to be a potential risk predictor and therapeutic target for cognitive impairment. However, more high-quality studies are needed to further investigate the dose relationship between circulating TMAO concentrations and cognitive impairment.

**Systematic review registration:**

PROSPERO, identifier: CRD42023464543.

## 1 Introduction

With an increasing aging population, cognitive impairment has become a global health problem (Winblad et al., 2004). Cognitive impairment is defined as a gradual decline in memory or other cognitive functions that does not affect the ability to perform daily living tasks and does not meet the diagnostic criteria for dementia (Petersen et al., 2018). According to the Global Burden of Diseases, Injuries, and Risk Factors Study (GBD) 2016 Dementia Collaborators study, the number of dementia cases increased globally by 117% between 1990 and 2016, making it the fifth leading cause of death worldwide (Global, Regional, and National Burden of Alzheimer's Disease and Other Dementias, 2019). Alzheimer Association Reports also reported that the number of Alzheimer’s deaths increased by more than 145% between 2000 and 2019, accompanied by high healthcare costs (Lv et al., 2023). However, early identification and intervention in cognitive impairment can effectively prevent the development of incurable neurodegenerative diseases.

Trimethylamine oxide (TMAO) is a metabolite synthesized in the liver from dietary precursors that are metabolized into intermediate products by intestinal microbiota (Abbasi, 2019). The precursors, such as choline, L-carnitine, and betaine, are obtained from red meat, eggs, milk, and fish. These precursors are metabolized to trimethylamine (TMA) by the gut microbiota. Subsequently, TMA is oxidized to TMAO by flavin-containing monooxygenase in the liver (Koeth et al., 2019; Wu et al., 2019; Li et al., 2022). Finally, TMAO can be released into the circulatory system and act in the brain through the blood–brain barrier (BBB).

Some studies have demonstrated the promoting effect of TMAO on cognitive impairment. TMAO can mediate pathological mechanisms, including the activation of inflammatory signaling pathways (Deng et al., 2022), endoplasmic reticulum stress (Wang et al., 2022), oxidative stress (Brunt et al., 2020), synaptic damage (Li et al., 2018; Liu J, et al., 2023), and can also induce astrocyte activation (Brunt et al., 2021), resulting in neuronal damage(Ji et al., 2022). However, conflicting results regarding the efficacy of TMAO have been reported. A clinical study found that plasma TMAO was not associated with cognition (Yaqub et al., 2024), consistent with the findings of Mendelian research (Zhuang et al., 2021). An *in vitro* study found that TMAO decreased the permeability of the tracer at typical levels (4 to 40 μM) and a significant reversal occurred at 100 times higher levels (4 mM), leading to increased permeability (Hoyles et al., 2021). Currently, there is no knowledge of the concentration ranges of TMAO in healthy versus diseased individuals. Therefore, there is a need to explore the effects of TMAO on cognitive impairment in humans and the differences in effects at different concentrations.

Meanwhile, since TMAO is a metabolite obtained from dietary sources, it is also worth exploring whether its effect on cognitive function is related to dietary structure. Previous studies have reported that the consumption of processed red meat increases TMAO concentration and affects cardiovascular and cerebrovascular diseases (Spence, 2018). However, another study found that TMAO was associated with healthy dietary intake, rather than meat, processed meat, and dairy products (Costabile et al., 2021). Therefore, our study sought to address the possible role of TMAO dietary precursor substances such as choline and betaine in cognitive impairment.

In this systematic review and meta-analysis, we aimed to evaluate the association between circulating TMAO levels and their precursors and the prevalence of cognitive impairment, providing new predictors and targets for the clinical prediction and prevention of cognitive impairment.

## 2 Materials and methods

### 2.1 Systematic review protocol and registration

This study was conducted based on the recommendations of the Preferred Reporting Items for Systematic Reviews (PRISMA) statement (Liu et al., 2019). The review agreement is registered in PROSPERO under the number CRD42023464543.

### 2.2 Literature search

We searched PubMed, Embase, and Web of Science databases, with the main search terms being “Cognitive Dysfunction,” “Cognitive Impairment,” “Cognitive Disorder,” “Mild Cognitive Impairment,” “MCI,” “cognitive decline,” “trimethyloxamine,” “trimethylamine N-oxide,” “TMAO,” “Carnitine,” “Betaine,” and “choline.” In the initial screening stage, references from previous systematic reviews and meta-analyses on similar topics were reviewed for supplementary purposes. In the full-text screening stage, the references from eligible articles were browsed for supplementation. No other published studies were identified. The specific retrieval strategy is presented in Supplementary Tables 1–3. The database retrieval period ranged from the establishment of the database to 14 September 2024, and the language was limited to English.

### 2.3 Inclusion and exclusion criteria

Inclusion criteria comprise: (1) study types include cohort, cross-sectional, or case–control studies. (2) Exposure factors are TMAO, choline, betaine, and carnitine; at least two categories of TMAO outcomes should be reported for a two-class meta-analysis, while at least three categories warrant dose–response meta-analysis. (3) The study population involves patients with cognitive impairment who meet diagnostic criteria, regardless of age, sex, or ethnicity. (4) TMAO should be reported concerning cognitive impairment patients, presented as the highest and lowest hazard ratios (HRs), relative risks (RRs), or odds ratios (ORs) with corresponding 95% confidence intervals (CIs). Additionally, we excluded duplicate publications, studies with inadequate data, and inconsistent study types, including reviews, systematic reviews, animal experiments, reports, letters, and case reports.

### 2.4 Study selection and data extraction

Two authors (CY.Long & ZH.Li) independently searched, screened, and cross-checked the literature. When there was a disagreement, the third author (RS.Yue) judged and concluded. The first screening was completed by browsing the titles and abstracts of the articles, and inclusion was determined by reading the full text. After screening the literature, two authors (CY.Long & ZH.Li) independently extracted data using a predesigned data extraction format that included information on the first author’s name, publication year, country, study design, underlying disease, source of exposure factors, cognitive impairment diagnostic scale name, 95%CI, TMAO dose, number of participants and cases, and study results.

### 2.5 Quality evaluation

The quality of each study was independently assessed by two authors (CY.Long & ZH.Li), and all discrepancies were resolved through consultation with the third author (RS.Yue). The Newcastle-Ottawa Scale (NOS) is used to assess the quality of cohorts and case–control studies (Wells and O’connor, 2012) and is evaluated by a three-module, eight-item approach, where studies with a total score of ≥6 are considered to be of high quality (Zeng et al., 2021). We conducted cross-sectional studies using the Agency for Healthcare Research and Quality (AHRQ) checklist (Rostom et al., 2004). If the answer was yes, the item score was 1; if the answer was no or unclear, the score was 0. A final score of 0–3 indicates that a study is of low quality, 4–7 indicates medium quality, and ≥ 8 indicates high quality.

### 2.6 Statistical analysis

This study used the “meta” software package in Rstudio 4.3.1 software for statistical analysis, and two-tailed *p* < 0.05 suggested that the difference was statistically significant.

First, we compared the prevalence of cognitive impairment between the highest and lowest TMAO levels and their precursors. OR and 95%CI were used to estimate the combined effects. Heterogeneity between the studies was assessed using the I^2^ statistical parameter. When there was significant heterogeneity (> 50%) in the fixed-effects model, meta-analysis was performed using a random-effects model (Foreman et al., 2021). Subgroup analyses were performed according to country, age, sex, underlying disease, diagnostic criteria, type of study, and quality of literature to explain potential confounding factors and their impact on cognitive impairment.

Second, we screened the included literature containing three or more TMAO categories for dose–response analysis. If the median or average concentration of circulating TMAO was not reported in this study, we switched to using the median of each category. If the boundary between the lowest and highest categories was open, the midpoint of the category was estimated by assuming that the interval was the same as that of the closest category. For each study, the lowest circulating TMAO concentration was defined as the reference dose. Linear and nonlinear correlations were checked using a random effects dose–response meta-analysis. A restricted cubic spline with 3 sections was used to calculate the study-specific OR estimate for every 1 μmol/L increase in TMAO concentration.

Finally, we evaluated the potential publication bias using Egger’s test. The robustness of the results was evaluated through a sensitivity analysis using a one-by-one division method.

## 3 Results

### 3.1 Literature screening

**Figure 1:**
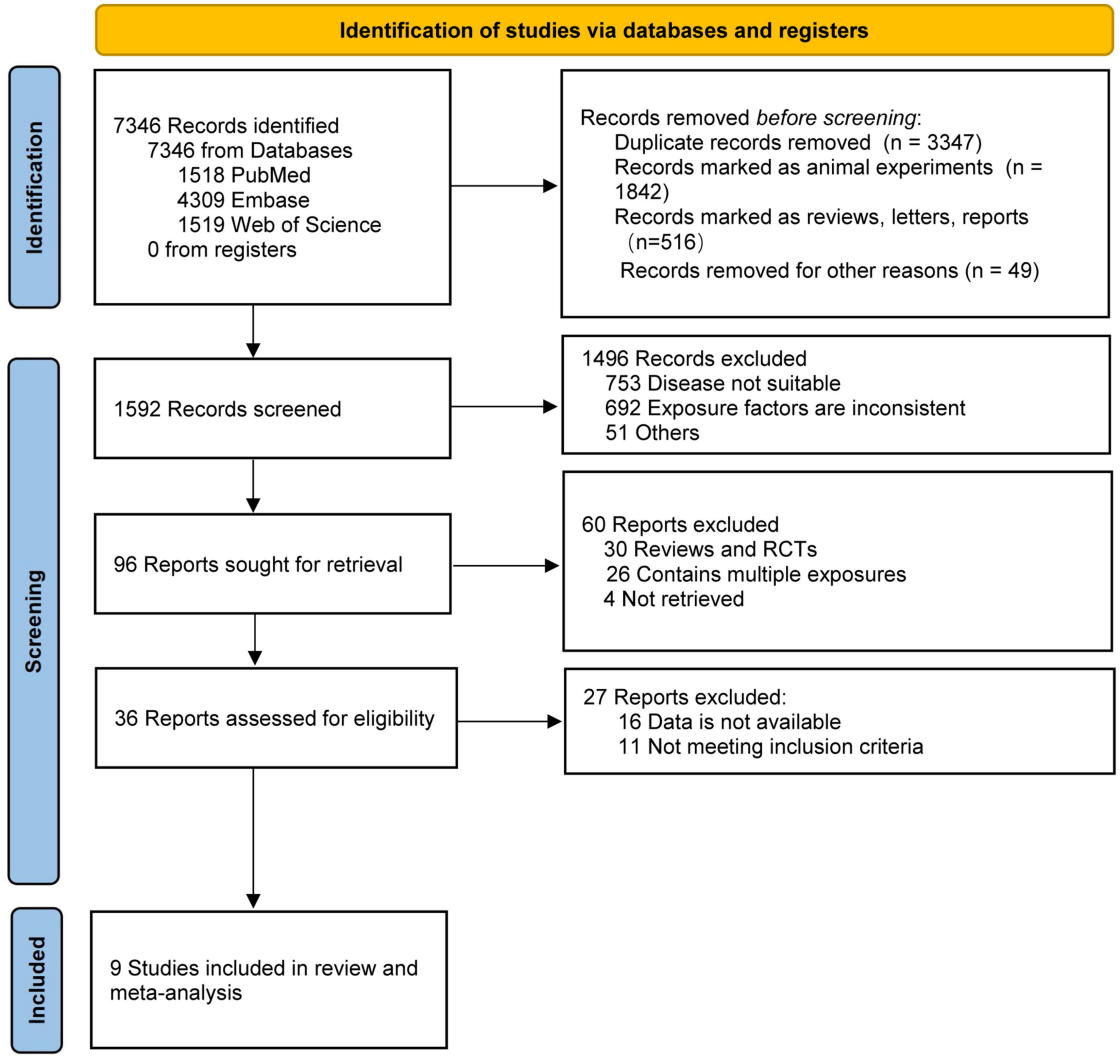
Meta-analysis flow chart.

Overall, 7,346 reports were initially retrieved. Duplicates were excluded by browsing titles, and studies such as animal experiments, reviews, letters, reports, etc., leaving 1,592 studies to continue browsing abstracts. After excluding articles that did not meet the criteria for nerfing, such as disease, exposure factors, and article format, 36 remained for full-text browsing. Finally, nine articles (Zhu et al., 2019; Liu et al., 2021; Zhong et al., 2021; Buawangpong et al., 2022; de Oliveira Otto et al., 2022; Flores-Torres et al., 2022; Xu et al., 2022; Wang et al., 2023; Shih et al., 2024) were screened for statistical analysis, including six cohort studies (Zhu et al., 2019; Zhong et al., 2021; de Oliveira Otto et al., 2022; Flores-Torres et al., 2022; Wang et al., 2023; Shih et al., 2024) and three cross-sectional studies (Liu et al., 2021; Buawangpong et al., 2022; Xu et al., 2022), with a total of 82,246 participants. A flowchart of the literature search and study selection processes is shown in Figure 1.

### 3.2 Literature features and quality evaluation

The basic characteristics of the included studies are presented in Table 1. Among these, five were from China (Zhu et al., 2019; Liu et al., 2021; Zhong et al., 2021; Xu et al., 2022; Wang et al., 2023), two from the United States (de Oliveira Otto et al., 2022; Flores-Torres et al., 2022), one from China Taiwan (Shih et al., 2024), and one from the Thailand (Buawangpong et al., 2022). There were six studies involving TMAO (Zhu et al., 2019; Zhong et al., 2021; Buawangpong et al., 2022; de Oliveira Otto et al., 2022; Xu et al., 2022; Wang et al., 2023), five on choline [three studies on dietary sources (Liu et al., 2021; Flores-Torres et al., 2022; Shih et al., 2024), two on plasma sources (Zhong et al., 2021; de Oliveira Otto et al., 2022; Shih et al., 2024)], and two on betaine (Zhong et al., 2021; de Oliveira Otto et al., 2022). Most literature reports baseline population information (Zhu et al., 2019; Liu et al., 2021; Zhong et al., 2021; Buawangpong et al., 2022; de Oliveira Otto et al., 2022; Xu et al., 2022; Wang et al., 2023). Most of the literature has adjusted for confounders (Zhu et al., 2019; Liu et al., 2021; Zhong et al., 2021; Buawangpong et al., 2022; de Oliveira Otto et al., 2022; Wang et al., 2023). In TMAO-related studies, the average TMAO circulating concentration was 2.63–8.5 μmol/L. The highest circulating concentration of TMAO is greater than 4.31 μmol/L. All studies specified particular detection methods.

**Table 1:** Basic characteristics.

Author (reference)	Year	Country	Study design	Age*, y	males, %	Population	Exposure	measurement method of exposure	Source of exposure	Diagnosis of CI	Participants, n	Study period	Hypertension, n (%)	Diabetes, n (%)	Drinking, n (%)	Coronary heart disease, n(%)	Adjusted confounders
Zhu (Zhu et al., 2019)	2019	China	cohort	67.1 ± 11.0	54.3	stroke	TMAO	HPLC–MS/MS	blood	MMSE	256	Jan 2017–Dec 2017	148 (57.8)	71 (27.7)	92 (35.9)	28 (10.9)	Age, education level, hypertension, diabetes, recurrent stroke, initial NIHSS score, white matter lesions, lowdensity lipoprotein, Hs-CRP, and homocysteine leve
Liu (Liu et al., 2021)	2021	China	cross sectional	NR	49.01	no disease restrictions	Choline	Questionnaire	dietary	WLS, AF, DSST	2,393	2011–2012, 2013–2014	1,502 (62.8)	559 (23.4)	1,666 (69.6)	N/A	Age, gender, BMI, alcohol consumption, and hypertension;
Zhong (Zhong et al., 2021)	2021	China	cohort	60 ± 10.5	70.19	stroke	TMAO, Choline, Betaine	UPLC–MS/MS	blood	MMSE, MoCA	617	Aug 2009–May 2013	475 (77.0)	104 (16.9)	N/A	66 (10.7)	Time from onset to randomization, admission NIHSS score, systolic BP, fasting plasma glucose, estimated glomerular filtration rate, medical history, use of antihypertensive and lipid-lowering medications, ischemic stroke subtype, and randomized treatment.
Nida (Buawangpong et al., 2022)	2022	Thailand	cross sectional	64 ± 8.4	45.49	cardiovascular high risk	TMAO	LC–MS/MS	blood	MoCA	233	Apr 2011–Mar 2014	195 (83.7)	156 (67.0)	N/A	N/A	Age, gender, health care service scheme, history of smoking, metabolic syndrome, and history of the established CV event.
Marcia (de Oliveira Otto et al., 2022)	2022	U.S.	cohort	71.6 ± 4.8	35	no disease restrictions	TMAO, Choline, Betaine	LC–MS/MS	blood	3MSE, IQCODE, TICS	3,178	1989–1990, 1992–1993	N/A	N/A	N/A	N/A	Red meat intake, fish, total energy consumption, eGFR, prevalent CHD, atrial fibrillation and heart failure.
Xu (Xu et al., 2022)	2022	China	cross sectional	64 (57.8–69)	51.78	T2DM	TMAO	HPLC–MS/MS	blood	MoCA	253	Jan 2018–Dec 2020	75 (29.6)	253 (100)	62 (24.5)	N/A	N/A
Wang (Wang et al., 2023)	2023	China	cohort	77.40 ± 7.88	51.6	TIA	TMAO	LC-MS/MS	blood	MMSE, MoCA, IQCODE	310	Jan 2020–July 2021	180 (58.1)	86 (27.7)	N/A	26 (8.4)	Age, sex, years of education, baseline NIHSS, intracranial atherosclerosis stenosis, Fazekas score, cortical microinfarcts and focal cerebral hypoperfusion.
Torres (Flores-Torres et al., 2022)	2022	U.S.	cohort	N/A	N/A	no disease restrictions	Choline	Questionnaire	dietary	N/A	77,501	2012–2014,2008–2012	N/A	N/A	N/A	N/A	N/A
Shih (Shih et al., 2024)	2024	Taiwan, China	Case- cohort	N/A	N/A	no disease restrictions	Choline	Questionnaire	dietary	MMSE	154	2019–2024	N/A	N/A	N/A	N/A	N/A

CI, cognitive impairment; T2DM, type 2 diabetes mellitus; TIA, transient ischemic attack; TMAO, Trimethylamine oxide; HPLC-MS/MS, High performance liquid chromatography–tandem mass spectrometry; UPLC-MS/MS, Ultra Performance Liquid Chromatography–tandem mass spectrometry; LC–MS/MS, Liquid chromatography–tandem mass spectrometry; MMSE, Mini-Mental State Examination; WLS, the Consortium to Establish a Registry for Alzheimer’s Disease (CERAD) Word Learning subset; AF, the Animal Fluency test; DSST, the Digit Symbol Substitution Test; MoCA, Montreal Cognitive Assessment; IQCODE, Informant Questionnaire on Cognitive Decline in the Elderly; N/A, Not Applicable; TICS, the Telephone Interview for Cognitive Status.

Six cohort studies were assessed using the NOS (Zhu et al., 2019; Zhong et al., 2021; de Oliveira Otto et al., 2022; Flores-Torres et al., 2022; Wang et al., 2023), of which four scored six or above (Zhu et al., 2019; Zhong et al., 2021; de Oliveira Otto et al., 2022; Wang et al., 2023; Shih et al., 2024), one scored five (Flores-Torres et al., 2022), and one scored four(Shih et al., 2024). Three cross-sectional studies were evaluated using the AHRQ checklist (Liu et al., 2021; Buawangpong et al., 2022; Xu et al., 2022), all scoring between four and seven. Further details are available in Supplementary material 1.

### 3.3 Meta-analysis of two types of comparison between circulating TMAO concentration and prevalence of cognitive impairment

**Figure 2:**
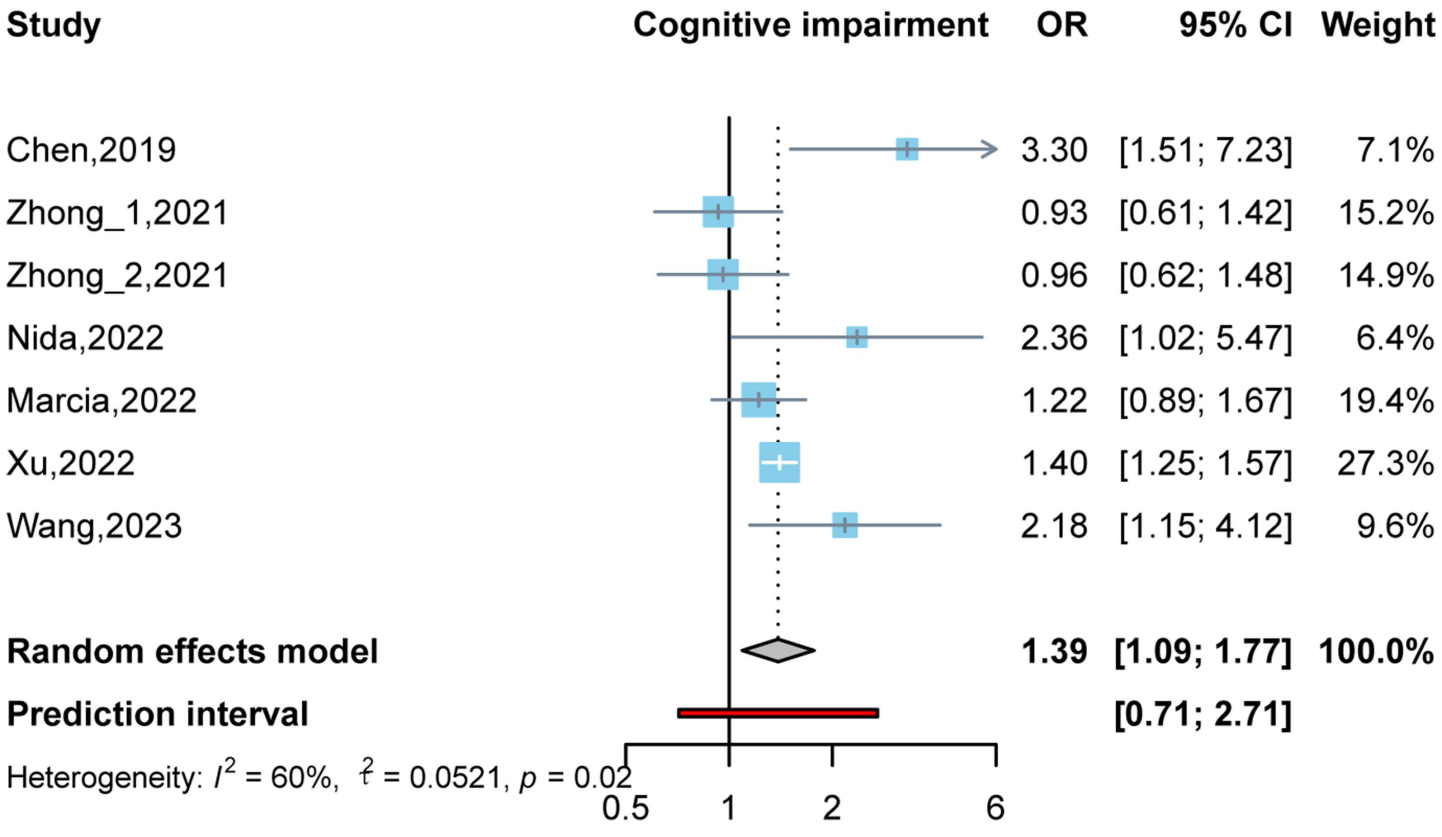
Odds ratio and 95% confidence interval of plasma trimethylamine oxide (TMAO) levels for cognitive impairment.

By conducting two meta-analyses, we compared the association between circulating TMAO concentration and the OR of cognitive impairment. As shown in Figure 2, there were six studies (Zhu et al., 2019; Zhong et al., 2021; Buawangpong et al., 2022; de Oliveira Otto et al., 2022; Xu et al., 2022; Wang et al., 2023) involving 1,766 participants. Comparing the prevalence of cognitive impairment between the highest and lowest concentrations of TMAO categories, it was found that higher TMAO concentrations were associated with higher prevalence of cognitive impairment (OR: 1.39, 95%CI: 1.09–1.77, *p* < 0.05, I^2^:60%, random effects model, Figure 2). Three articles (Zhu et al., 2019; Zhong et al., 2021; Wang et al., 2023) were used to assess whether there was a dose–response relationship between circulating TMAO concentrations and the prevalence of cognitive impairment. Linear and nonlinear dose–response meta-analyses were performed; however, the results were not significant (*P*-nonlinearity = 0.205, *P*-linearity = 0.059). More details are shown in Supplementary Figures 1, 2.

### 3.4 Subgroup analysis

Due to the high heterogeneity of the studies (Zhu et al., 2019; Zhong et al., 2021; Buawangpong et al., 2022; de Oliveira Otto et al., 2022; Xu et al., 2022; Wang et al., 2023), a subgroup analysis was performed to assess the impact of the underlying population, study type, age, sex ratio, cognitive function diagnostic scale, and sample size on the cognitive function results. As four of the included articles were from China (Zhu et al., 2019; Zhong et al., 2021; Xu et al., 2022; Wang et al., 2023), one was from the United States (de Oliveira Otto et al., 2022), and one was from Thailand (Buawangpong et al., 2022), a subgroup analysis was not performed at the national level. Similarly, among the included studies, only one study on TMAO was derived from serum (Xu et al., 2022), while the rest were derived from plasma (Zhu et al., 2019; Zhong et al., 2021; Buawangpong et al., 2022; de Oliveira Otto et al., 2022; Wang et al., 2023). Therefore, a subgroup analysis was not performed; instead, the effect on the outcome of each study was observed using the one-by-one elimination method in the sensitivity analysis. The results of subgroup analysis suggest that these factors are not significant influences on heterogeneity (Table 2).

**Table 2:** Subgroup analysis.

Subgroups	Studies, n [references]	OR	95%CI	*P* between group	I*^2^*, %	*P* heterogeneity
All	6 (Zhu et al., 2019; Zhong et al., 2021; Buawangpong et al., 2022; de Oliveira Otto et al., 2022; Xu et al., 2022; Wang et al., 2023)	1.39	1.09–1.77		60	0.02
Population						
Stroke	2 (Zhu et al., 2019; Zhong et al., 2021)	1.31	0.70–2.45	0.78	77	0.01
Others	4 (Buawangpong et al., 2022; de Oliveira Otto et al., 2022; Xu et al., 2022; Wang et al., 2023)	1.44	1.19–1.73		26	0.26
Design						
cohort study	4 (Zhu et al., 2019; Zhong et al., 2021; de Oliveira Otto et al., 2022; Wang et al., 2023)	1.37	0.94–1.99	0.68	67	0.02
cross sectional study	2 (Buawangpong et al., 2022; Xu et al., 2022)	1.53	1.05–2.23		31	0.23
Olds						
>65	3 (Zhu et al., 2019; de Oliveira Otto et al., 2022; Wang et al., 2023)	1.9	1.04–3.48	0.20	71	0.03
<65	3 (Zhong et al., 2021; Buawangpong et al., 2022; Xu et al., 2022)	1.23	0.91–1.66		60	0.06
Males (%)						
>50	4 (Zhu et al., 2019; Zhong et al., 2021; Xu et al., 2022; Wang et al., 2023)	1.4	1.01–1.94	0.83	69	0.01
<50	2 (Buawangpong et al., 2022; de Oliveira Otto et al., 2022)	1.51	0.82–2.75		52	0.15
Diagnose						
MMSE	2 (Zhu et al., 2019; Zhong et al., 2021)	1.68	0.49–5.79	0.89	87	<0.01
MoCA	3 (Zhong et al., 2021; Buawangpong et al., 2022; Xu et al., 2022)	1.34	0.95–1.87		54	0.11
Others	2 (de Oliveira Otto et al., 2022; Wang et al., 2023)	1.52	0.88–2.64		61	0.11
Participants, n						
< 250	4 (Zhong et al., 2021; Buawangpong et al., 2022; Xu et al., 2022; Wang et al., 2023)	1.88	0.71–4.94	0.50	81	0.02
> 250	2 (Zhu et al., 2019; de Oliveira Otto et al., 2022)	1.32	0.99–1.76		58	0.05

OR, odds ratio; 95%CI, corresponding 95% confidence intervals; MMSE, Mini-Mental State Examination; MoCA, Montreal Cognitive Assessment.

### 3.5 Sensitivity analysis

A sensitivity analysis was conducted to confirm the correlation between TMAO concentrations and the prevalence of cognitive impairment. The results were validated by sequentially removing one included study using a random effects model. As shown in Table 3, the combined results of the remaining studies did not change direction after each literature was excluded, suggesting that the results are robust.

**Table 3:** Sensitivity analysis.

Study removed (reference)	OR	95%CI	*p* value	I*^2^*, %
Zhong 2021 (Zhong et al., 2021)	1.617	1.234–2.120	0.005	52.9
Wang 2023 (Wang et al., 2023)	1.324	1.030–1.701	0.028	61.1
Xu 2022 (Xu et al., 2022)	1.454	1.016–2.081	0.041	64.9
Marcia 2022 (de Oliveira Otto et al., 2022)	1.468	1.076–2.002	0.016	65.1
Nida 2022 (Buawangpong et al., 2022)	1.339	1.046–1.715	0.021	62.4
Zhu 2019 (Zhu et al., 2019)	1.294	1.045–1.603	0.018	50.1

OR, odds ratio; 95%CI, corresponding 95% confidence intervals.

### 3.6 Publication bias

No evidence of publication bias was found using the Egger test in the highest and lowest TMAO categories (*p* = 0.703) or in circulating TMAO (*p* = 0.147).

### 3.7 Meta-analysis of the correlation between TMAO precursors and cognitive function

This review mainly retrieved studies related to choline levels in the plasma (Zhong et al., 2021; de Oliveira Otto et al., 2022) and diet (Liu et al., 2021; Flores-Torres et al., 2022; Shih et al., 2024), as well as betaine in plasma (Zhong et al., 2021; de Oliveira Otto et al., 2022); however, no studies related to L-carnitine have been performed. The results indicated that the above results were not statistically significant (dietary choline: OR: 0.93, 95%CI: 0.78–1.10, *p* = 0.385, I^2^:68%, plasma choline: OR: 0.65, 95%CI: 0.41–1.02, *p* = 0.063, I^2^:76%, plasma betaine: OR: 0.74, 95%CI: 0.52–1.05, *p* = 0.094, I^2^:61%). See details in Supplementary Figures 3–5.

## 4 Discussion

### 4.1 Potential mechanism underlying the role of TMAO in cognitive impairment

*In vitro* and *in vivo* studies have found that the negative effects of pathologically elevated TMAO on cognitive impairment can be explained by the following mechanisms.

First, TMAO can affect the central neuronal structure. TMAO impairs neuronal synaptic plasticity through the mTOR/P70S6K/4EBP1 pathway (Liu J, et al., 2023), leading to cognitive impairment. TMAO can also directly reduce the number and density of synapses in Alzheimer’s mice (Zarbock et al., 2022). Studies have been conducted to restore cognitive function by targeting TMAO. The ZeXieYin Formula restores synaptic plasticity and alleviates cognitive impairment by targeting TMAO through the mTOR signaling pathway (Liu J, et al., 2023).

Second, TMAO impairs the structure and function of BBB, which likewise leads to cognitive impairment. Brain microvascular endothelial cells are the main components of the BBB. It has been found that TMAO is closely associated with platelet hyperactivity and lipid metabolism disorders, and can regulate vascular endothelial function (Jin et al., 2024). In patients with chronic kidney disease, correlation analyses of TMAO with BBB markers brain-derived neurotrophic factor (BDNF) and neuron-specific enolase (NSE) showed significant negative and positive correlations and possible impaired BBB integration (Hernandez et al., 2022).

Third, TMAO can also trigger CNS inflammation through the NLRP3 inflammatory signaling pathway (Ge et al., 2023), oxidative stress (Deng et al., 2022), and endoplasmic reticulum stress (Wang et al., 2022), when it is in a state of illness. Subsequently, these inflammatory responses can induce glial and vascular cell dysfunction, ultimately triggering neuronal injury. For example, TMAO promotes a microglial pro-inflammatory phenotype and triggers phagocytic disorders (Janeiro et al., 2023). High concentrations of serum TMAO can cause reactive astrocytes and increase levels of TNF-*α* and IL-1β (Qiao et al., 2023).

### 4.2 Effects of TMAO concentration on cognitive impairment and relative cognitive domains

This study also noted the effect of TMAO dosage on cognitive impairment. Researchers have found that the fasting concentration of TMAO in the plasma of the general population ranges from 3.6 to 3.7 μmol/L (Kühn et al., 2017). One research constructed animal models based on human circulating TMAO concentrations, and found that TMAO (1.8 mg/kg) had an acute beneficial effect on the BBB at physiological concentrations (Hoyles et al., 2021). However, the relationship between TMAO levels and cognitive impairment under pathological conditions remains unclear. In the six articles we included (Zhu et al., 2019; Zhong et al., 2021; Buawangpong et al., 2022; de Oliveira Otto et al., 2022; Xu et al., 2022; Wang et al., 2023), the mean median concentration of TMAO was 2.63–8.5 μmol/L. Chen et al. found that plasma TMAO levels >7.4 μmol/L were an independent risk factor for PSCI (Zhu et al., 2019), while Xu et al. found that serum TMAO levels >14.14 μmol/L significantly increased the risk of MCI in type 2 diabetes mellitus (T2DM) patients (Xu et al., 2022). The effect of the TMAO concentration on cognitive impairment may be regulated by an underlying disease. In the dose–response relationship in this study, no significant correlation was found in either the linear or nonlinear dose–response relationships. However, in the six included articles, all the pathological concentrations of TMAO were greater than 4.31 μmol/L, which may provide some support for the relationship between TMAO dose and cognitive dysfunction.

The cognitive domain refers to various aspects involved in an individual’s cognitive processes, typically encompassing executive function, processing speed, memory, attention, and language (Divandari et al., 2023). Since cognition does not uniformly depend on bodily functions (Clouston et al., 2013), it is crucial to examine whether TMAO and its concentration exert unique effects on specific domains. In the literature related to TMAO included in this study, most research explored its impact on global assessment through the MMSE (Zhu et al., 2019; Zhong et al., 2021) and MoCA (Zhong et al., 2021; Buawangpong et al., 2022; Xu et al., 2022; Wang et al., 2023). Additionally, Wang and colleagues found that higher TMAO levels correlated with increased vulnerability to impairments in executive and memory functions (Wang et al., 2023). Moreover, Marcia C. de Oliveira Ottow and associates assessed cognitive impairment using the Modified Mini-Mental State Examination (3MSE), which evaluates various cognitive domains, including memory, orientation, calculation, and verbal fluency (de Oliveira Otto et al., 2022). In animal studies, research confirmed TMAO’s impact on hippocampal synaptic plasticity, which similarly indicated detrimental effects on learning and memory functions (Zhou et al., 2023).

### 4.3 The potential applications of TMAO in therapeutic contexts

Currently, researchers have identified TMAO as a potential biomarker for disease and a new therapeutic target (Janeiro et al., 2018), primarily focusing on cardiovascular implications. For instance, treatment with rosuvastatin can lower plasma TMAO levels in patients with atherosclerotic cardiovascular disease, leading to improved lipid metabolism (Xiong et al., 2022). Ma et al. discovered through animal studies that berberine can reduce TMAO production and address atherosclerosis (Ma et al., 2022). In terms of cognitive function, the TMAO inhibitor 3,3-Dimethyl-1-butanol (DMB) can restore cognitive deficits in aging mouse models by alleviating neuroinflammation and enhancing insulin resistance (Lanz et al., 2022). Additionally, Jing Liu et al. found that ZeXieYin Formula mitigates TMAO-induced cognitive impairments by repairing synaptic plasticity damage (Liu J, et al., 2023). Further clinical studies targeting TMAO to treat cognitive dysfunction remain essential.

### 4.4 The effects of the precursors of TMAO on cognitive impairment

Betaine, choline, and L-carnitine, which are precursors of TMAO from dietary sources, may affect the circulating concentration of TMAO. Although several studies have suggested a correlation between TMAO and dietary structure, data on the relationship between dietary compounds such as choline and TMAO are not clear. One study found an association of homeostasis between TMAO, betaine, and choline, with the study population of “high betaine + TMAO” having a protective effect against cardiovascular disease (Huang et al., 2023). Our research retrieved four articles on choline from hemorrhagic plasma (Zhong et al., 2021; de Oliveira Otto et al., 2022) and diet (Liu et al., 2021; Flores-Torres et al., 2022), as well as two on betaine from plasma (Zhong et al., 2021; de Oliveira Otto et al., 2022), and did not find any association with cognitive impairment. This is similar to the conclusion of another meta-analysis of the relationship between TMAO and its precursors and stroke (Liu D, et al., 2023). Due to the insufficient literature, more research is needed to explore the effects of choline and betaine on cognitive function.

### 4.5 Advantages and limitations

This study has the following advantages: (1) We conducted a comprehensive search and included TMAO and its precursors on cognitive impairment following a standardized protocol, and the included studies were of high quality, most of which were adjusted for covariates to exclude confounding factors. (2) The included literature focuses on 2019–2023, ensuring contemporaneity with current public needs. (3) We found that TMAO may be a risk predictor and therapeutic target for cognitive impairment and is not due to dietary sources of choline and betaine intake but is more likely due to underlying diseases resulting from abnormal synthesis in the body.

Meanwhile, study has the following limitations: (1) There is a paucity of clinical studies, and more multicenter, large-scale studies are needed to assess the relationship between circulating TMAO concentrations and the risk of cognitive impairment. (2) Discussions about TMAO and cognitive impairment frequently emphasize broad evaluations, neglecting specific cognitive domains. A deeper inquiry is needed to uncover TMAO’s influence on particular cognitive domains. (3) The studies surveyed primarily stem from China, raising concerns about potential bias. (4) Diagnosing cognitive dysfunction largely depends on questionnaire-based general assessments, lacking advancements in computer-assisted diagnostics, which reduces both sensitivity and reproducibility. (5) Participants in the reviewed studies frequently present with pre-existing health conditions that may introduce bias in the results.

## 5 Conclusion

This is the first study to analyze the relationship between TMAO and its precursors, and the risk of cognitive impairment in a global population. This study revealed a significant positive correlation between TMAO and the prevalence of cognitive impairment, particularly affecting global cognitive assessment, and to some extent, memory and executive functioning. However, no relationship was found between cognitive impairment and specific doses. In addition, our study found no evidence that dietary choline, plasma choline, or betaine (i.e., TMAO precursors) were associated with the incidence of cognitive impairment.

There are few clinical studies on the relationship between TMAO and its precursors and cognitive impairment and not enough focus on cognitive domains, researchers need to conduct more center-based clinical studies with large samples and assess the unique role of TMAO on specific cognitive domains to provide conclusive data.

## Data Availability

The original contributions presented in the study are included in the article/Supplementary material, further inquiries can be directed to the corresponding author.
