# Corrigendum: Association of trimethylamine oxide and its precursors with cognitive impairment: a systematic review and meta-analysis

**DOI:** 10.3389/fnagi.2024.1519363

**Published:** 2024-11-19

**Authors:** Caiyi Long, Zihan Li, Haoyue Feng, Yayi Jiang, Yueheng Pu, Jiajing Tao, Rensong Yue

**Affiliations:** ^1^Hospital of Chengdu University of Traditional Chinese Medicine, Chengdu, China; ^2^Chengdu University of Traditional Chinese Medicine, Chengdu, China

**Keywords:** trimethylamine oxide, TMAO, circulating concentration, cognitive impairment, meta-analysis

In the published article, there were errors in Figures 1, 2 and Tables 1, 2 as published. The order of the images in Figures 1 and 2 is reversed (the titles are correct, but the sequence of the images is incorrect). The first row of authors and references in Table 1, “Zhong (Zhong et al., 2021),” is incorrect. The correct citation should be “Zhu (Zhu et al., 2019)”. In Table 2, the references in the subgroup analysis for the group under 65, specifically “(Zhu et al., 2019; de Oliveira Otto et al., 2022; Wang et al., 2023)” are incorrect. They should be: “Zhong et al., 2021; Buawangpong et al., 2022; Xu et al., 2022.” The corrected Figures 1, 2 and Tables 1, 2 appear below.

**Figure 1:**
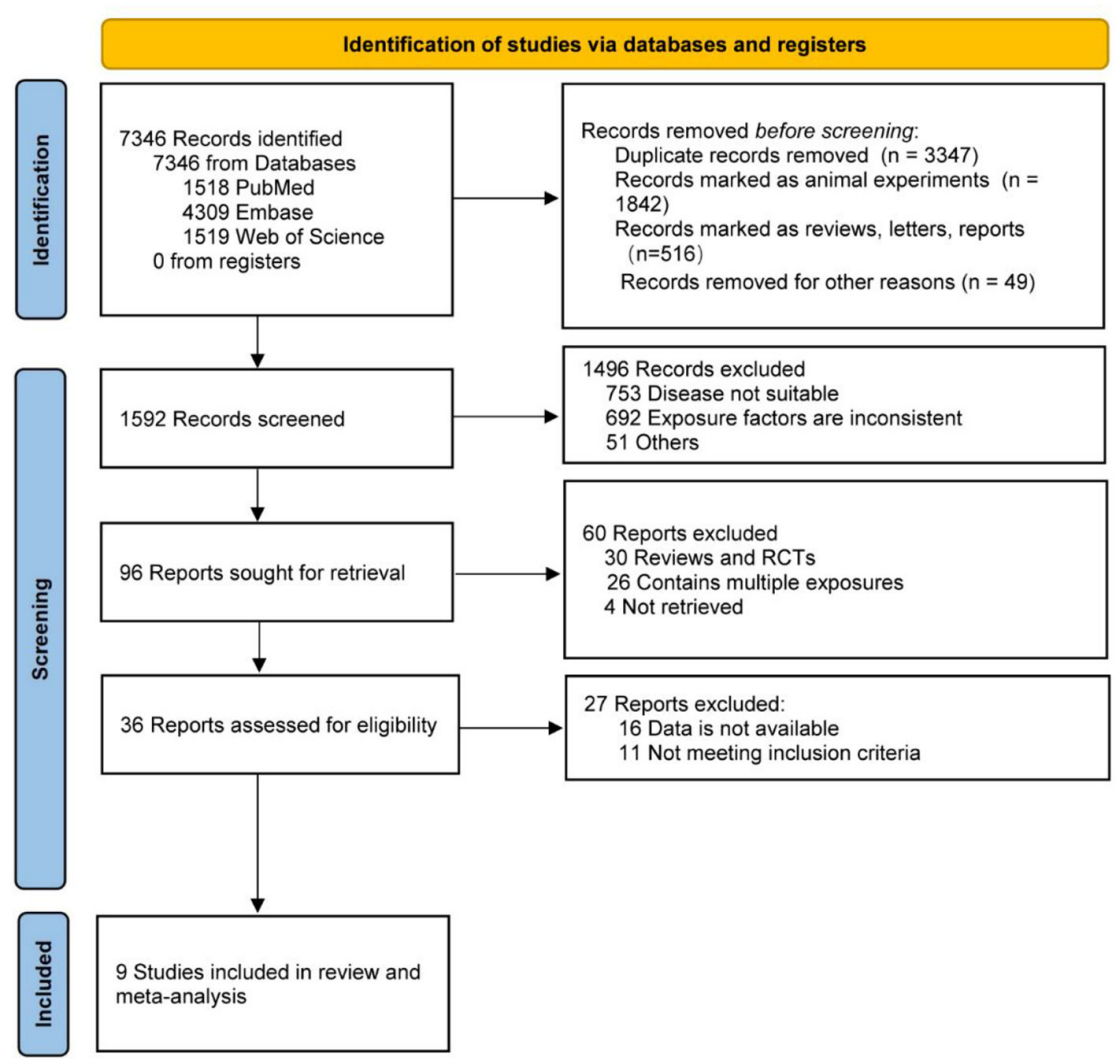
Meta-analysis flow chart.

**Figure 2:**
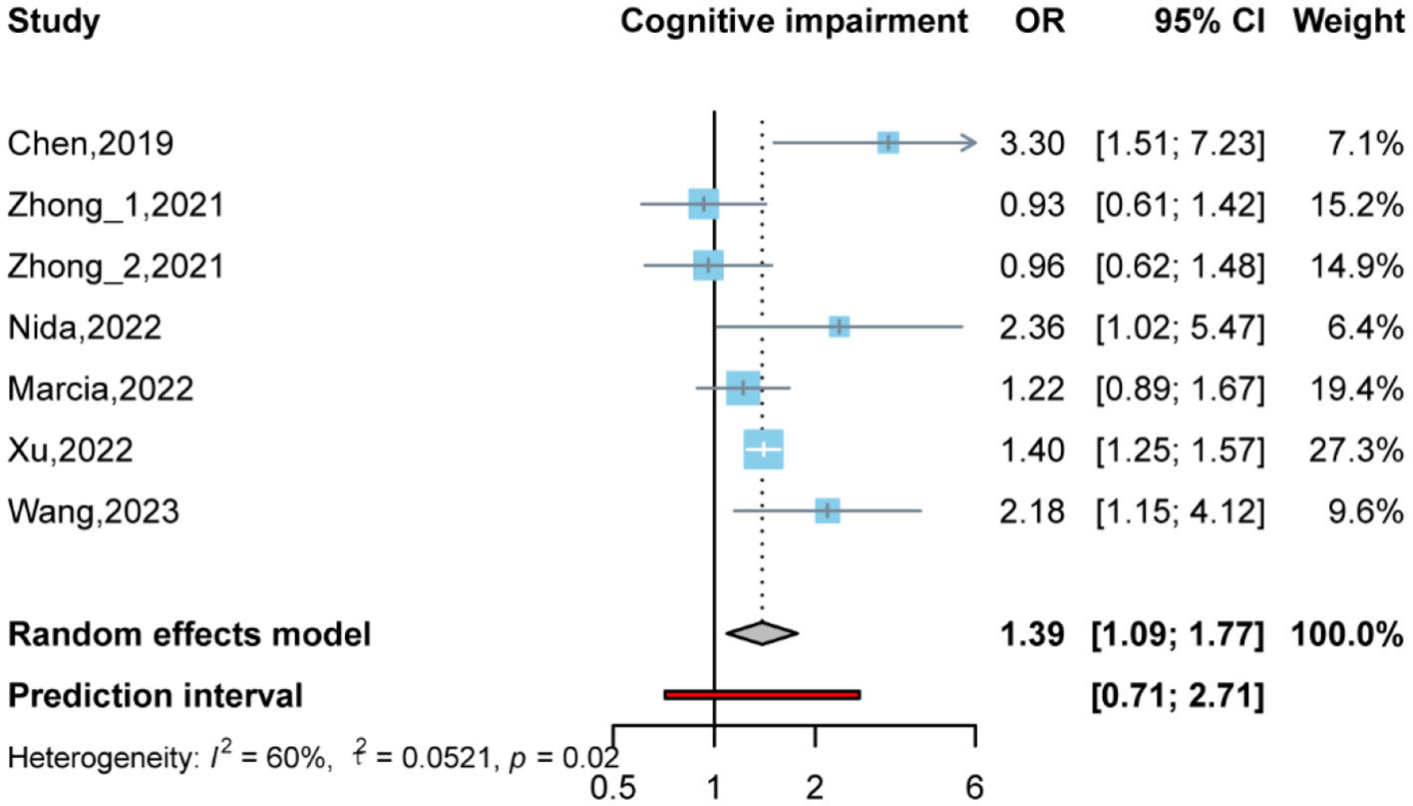
Odds ratio and 95% confidence interval of plasma trimethylamine oxide (TMAO) levels for cognitive impairment.

**Table 1:** Basic characteristics.

**References**	**Year**	**Country**	**Study design**	Age^*^, y	**Males, %**	**Population**	**Exposure**	**Measurement method of exposure**	**Source of exposure**	**Diagnosis of CI**	**Participants, *n***	**Study period**	**Hypertension, *n* (%)**	**Diabetes, *n* (%)**	**Drinking, *n* (%)**	**Coronary heart disease, *n* (%)**	**Adjusted confounders**
Zhu (Zhu et al., 2019)	2019	China	Cohort	67.1 ± 11.0	54.3	Stroke	TMAO	HPLC-MS/MS	Blood	MMSE	256	Jan 2017–Dec 2017	148 (57.8)	71 (27.7)	92 (35.9)	28 (10.9)	Age, education level, hypertension, diabetes, recurrent stroke, initial NIHSS score, white matter lesions, low density lipoprotein, Hs-CRP, and homocysteine leve;
Liu (Liu et al., 2021)	2021	China	Cross sectional	N/A	49.01	No disease restrictions	Choline	Questionnaire	Dietary	WLS, AF, DSST	2393	2011–2012, 2013–2014	1,502 (62.8)	559 (23.4)	1,666 (69.6)	N/A	Age, gender, BMI, alcohol consumption, and hypertension;
Zhong (Zhong et al., 2021)	2021	China	Cohort	60 ± 10.5	70.19	Stroke	TMAO, Choline, Betaine	UPLC-MS/MS	Blood	MMSE, MoCA	617	Aug 2009–May 2013	475 (77.0)	104 (16.9)	N/A	66 (10.7)	Time from onset to randomization, admission NIHSS score, systolic BP, fasting plasma glucose, estimated glomerular filtration rate, medical history, use of antihypertensive and lipid-lowering medications, ischemic stroke subtype, and randomized treatment.
Nida (Buawangpong et al., 2022)	2022	Thailand	Cross sectional	64 ± 8.4	45.49	cardiovascular high risk	TMAO	LC-MS/MS	blood	MoCA	233	Apr 2011–Mar 2014	195 (83.7)	156 (67.0)	N/A	N/A	Age, gender, health care service scheme, history of smoking, metabolic syndrome, and history of the established CV event.
Marcia (de Oliveira Otto et al., 2022)	2022	U.S.	Cohort	71.6 ± 4.8	35	No disease restrictions	TMAO, Choline, Betaine	LC-MS/MS	Blood	3MSE, IQCODE, TICS	3,178	1989–1990, 1992–1993	N/A	N/A	N/A	N/A	Red meat intake, fish, total energy consumption, eGFR, prevalent CHD, atrial fibrillation and heart failure.
Xu (Xu et al., 2022)	2022	China	Cross sectional	64 (57.8–69)	51.78	T2DM	TMAO	HPLC-MS/MS	Blood	MoCA	253	Jan 2018–Dec 2020	75 (29.6)	253 (100)	62 (24.5)	N/A	N/A
Wang (Wang et al., 2023)	2023	China	Cohort	77.40 ± 7.88	51.6	TIA	TMAO	LC-MS/MS	Blood	MMSE, MoCA, IQCODE	310	Jan 2020–July 2021	180 (58.1)	86 (27.7)	N/A	26 (8.4)	Age, sex, years of education, baseline NIHSS, intracranial atherosclerosis stenosis, Fazekas score, cortical microinfarcts and focal cerebral hypoperfusion.
Torres (Flores-Torres et al., 2022)	2022	U.S.	Cohort	N/A	N/A	No disease restrictions	Choline	Questionnaire	Dietary	N/A	77,501	2012–2014, 2008–2012	N/A	N/A	N/A	N/A	N/A
Shih (Shih et al., 2024)	2024	Taiwan, China	Case- cohort	N/A	N/A	No disease restrictions	Choline	Questionnaire	Dietary	MMSE	154	2019–2024	N/A	N/A	N/A	N/A	N/A

CI, cognitive impairment; T2DM, type 2 diabetes mellitus; TIA, transient ischemic attack; TMAO, Trimethylamine oxide; HPLC-MS/MS, High performance liquid chromatography-tandem mass spectrometry; UPLC-MS/MS, Ultra Performance Liquid Chromatography-tandem mass spectrometry; LC-MS/MS, Liquid chromatography-tandem mass spectrometry; MMSE, Mini-Mental State Examination; WLS, the Consortium to Establish a Registry for Alzheimer's Disease (CERAD) Word Learning subset; AF, the Animal Fluency test; DSST, the Digit Symbol Substitution Test; MoCA, Montreal Cognitive Assessment; IQCODE, Informant Questionnaire on Cognitive Decline in the Elderly; N/A, Not Applicable; TICS, the Telephone Interview for Cognitive Status.

**Table 2:** Subgroup analysis.

**Subgroups**	**Studies, *n* (references)**	**OR**	**95%CI**	***P* between group**	***I^2^*, %**	***P* heterogeneity**
All	6 (Zhu et al., 2019; Zhong et al., 2021; Buawangpong et al., 2022;de Oliveira Otto et al., 2022; Xu et al., 2022; Wang et al., 2023)	1.39	1.09–1.77		60	0.02
**Population**						
Stroke	2 (Zhu et al., 2019; Zhong et al., 2021)	1.31	0.70–2.45	0.78	77	0.01
Others	4 (Buawangpong et al., 2022;de Oliveira Otto et al., 2022; Xu et al., 2022; Wang et al., 2023)	1.44	1.19–1.73		26	0.26
**Design**						
Cohort study	4 (Zhu et al., 2019; Zhong et al., 2021;de Oliveira Otto et al., 2022; Wang et al., 2023)	1.37	0.94–1.99	0.68	67	0.02
Cross sectional study	2 (Buawangpong et al., 2022; Xu et al., 2022)	1.53	1.05–2.23		31	0.23
**Olds**						
>65	3 (Zhu et al., 2019;de Oliveira Otto et al., 2022; Wang et al., 2023)	1.9	1.04–3.48	0.20	71	0.03
< 65	3 (Zhong et al., 2021; Buawangpong et al., 2022; Xu et al., 2022)	1.23	0.91–1.66		60	0.06
**Males (%)**						
>50	4 (Zhu et al., 2019; Zhong et al., 2021; Xu et al., 2022; Wang et al., 2023)	1.4	1.01–1.94	0.83	69	0.01
< 50	2 (Buawangpong et al., 2022; de Oliveira Otto et al., 2022)	1.51	0.82–2.75		52	0.15
**Diagnose**						
MMSE	2 (Zhu et al., 2019; Zhong et al., 2021)	1.68	0.49–5.79	0.89	87	< 0.01
MoCA	3 (Zhong et al., 2021; Buawangpong et al., 2022; Xu et al., 2022)	1.34	0.95–1.87		54	0.11
Others	2 (de Oliveira Otto et al., 2022; Wang et al., 2023)	1.52	0.88–2.64		61	0.11
**Participants**, ***n***						
< 250	4 (Zhong et al., 2021; Buawangpong et al., 2022; Xu et al., 2022; Wang et al., 2023)	1.88	0.71–4.94	0.50	81	0.02
>250	2 (Zhu et al., 2019; de Oliveira Otto et al., 2022)	1.32	0.99–1.76		58	0.05

OR, odds ratio; 95% CI, corresponding 95% confidence intervals; MMSE, Mini-Mental State Examination; MoCA, Montreal Cognitive Assessment.

The authors apologize for this error and state that this does not change the scientific conclusions of the article in any way. The original article has been updated.

